# Time to administer polymyxin B hemoperfusion and hemodynamics in patients with septic shock requiring high-dose norepinephrine: a predetermined analysis of a prospective cohort study

**DOI:** 10.1186/s13054-025-05422-7

**Published:** 2025-05-09

**Authors:** Kyohei Miyamoto, Yu Kawazoe, Noriko Miyagawa, Hitoshi Yamamura, Yoshinori Ohta, Takuya Kimura, Yukitoshi Toyoda, Michihito Kyo, Tetsuya Sato, Masashi Kinjo, Masaki Takahashi, Junichi Maruyama, Hiroshi Matsuura, Kazunori Fukushima, Satoru Murata, Tomoya Okazaki, Tsuyoshi Suzuki, Toshihiro Sakurai, Gaku Takahashi, Tasuku Hanajima, Takeshi Morimoto

**Affiliations:** 1 Department of Emergency and Critical Care Medicine, Wakayama Medical University, 811-1 Kimiidera, Wakayama, 641-8509 Japan; 2 Department of Emergency and Critical Care Medicine, National Hospital Organization Sendai Medical Center, Sendai, Japan; 3 Department of Emergency and Critical Care Medicine, Osaka Minato Central Hospital, Osaka, Japan; 4 Department of Emergency and Critical Care Medicine, National Hospital Organization Kyoto Medical Center, Kyoto, Japan; 5 Department of Emergency Medicine and Critical Care Medicine, Saiseikai Utsunomiya Hospital, Utsunomiya, Japan; 6 Department of Emergency and Critical Care Medicine, Saiseikai Yokohamashi Tobu Hospital, Yokohama, Japan; 7 Department of Emergency and Critical Care Medicine, Graduate School of Biomedical and Health Sciences, Hiroshima University, Hiroshima, Japan; 8 Department of Radiation Disaster Medicine, Research Institute for Radiation Biology and Medicine, Hiroshima University, Hiroshima, Japan; 9 Department of Emergency and Critical Care Medicine, Tohoku University Hospital, Sendai, Japan; 10 Division of Critical Care Medicine, Nara Prefecture General Medical Center, Nara, Japan; 11 Division of Acute and Critical Care Medicine, Department of Anesthesiology and Critical Care Medicine, Faculty of Medicine, Hokkaido University, Sapporo, Japan; 12 Department of Emergency Medicine and Critical Care, Fukuoka University Hospital, Fukuoka, Japan; 13 Osaka Prefectural Nakakawachi Emergency and Critical Care Center, Osaka, Japan; 14 Department of Emergency Medicine, Gunma University Graduate School of Medicine, Maebashi, Gunma Japan; 15 Department of Emergency and Critical Care Medicine, Ehime University Graduate School of Medicine, Toon, Japan; 16 Emergency Medical Center, Kagawa University Hospital, Kida, Japan; 17 Department of Emergency and Critical Care Medicine, Fukushima Medical University, Fukushima, Japan; 18 Department of Emergency and Critical Care Medicine, National Hospital Organization Kumamoto Medical Center, Kumamoto, Japan; 19 Department of Critical Care and Disaster Medicine, Iwate Medical University, Shiwa, Japan; 20 Trauma and Reconstruction Center, Shin-Yurigaoka General Hospital, Kawasaki, Japan; 21 Department of Data Science, Hyogo Medical University, Nishinomiya, Japan

**Keywords:** Septic shock, Polymyxin B hemoperfusion, Blood purification

## Abstract

**Background:**

Delayed administration of polymyxin B hemoperfusion (PMX-HP) for septic shock could diminish its efficacy in real-world clinical settings.

**Methods:**

BEAT-SHOCK (BEst Available Treatment for septic SHOCK) registry is a prospective registry consisting of 309 adult patients with septic shock requiring high-dose norepinephrine (≥ 0.2 μg/kg/min). This predetermined analysis included 82 patients treated with PMX-HP. They were grouped according to the median time from intensive care unit (ICU) admission to administration of PMX-HP: the early administration group (n = 40) and the late administration group (n = 42). The primary outcome was short-term hemodynamic status, including mean arterial pressure and vasoactive-inotropic score (VIS; calculated from doses of dopamine, dobutamine, norepinephrine, epinephrine, vasopressin, milrinone, and levosimendan) within 48 h after ICU admission.

**Results:**

The median time from ICU admission to administration of PMX-HP was 265 min (interquartile range [IQR]: 113–480). The median ages were 70 (IQR: 59–81) and 72 (IQR: 64–80) years (*P* = 0.77), and 21/40 (53%) and 25/42 (60%) patients were male (*P* = 0.52) in the early and late administration groups, respectively. The dose of norepinephrine at ICU admission was 0.33 (IQR: 0.24–0.47) and 0.30 (IQR: 0.22–0.34) μg/kg/min in the early and late administration groups, respectively (*P* = 0.17). Within 48 h after ICU admission, mean arterial pressure was significantly higher at 6 h and 8 h, and VIS was significantly lower at 8 h and thereafter in the early administration group. Within a 28-day period, there were 23 (IQR: 21–25) and 21 (IQR: 0–24) vasopressor/inotrope-free days (*P* = 0.027), and 18 (IQR: 1–23) and 14 (IQR: 0–19) ICU-free days (*P* = 0.025) in the early and late administration groups, respectively. The cumulative mortality at 90 days was 15.3% in the early administration group and 31.3% in the late administration group (adjusted hazard ratio 0.38; 95% confidence interval 0.13–1.09).

**Conclusions:**

In patients with septic shock, early administration of PMX-HP was associated with higher mean arterial pressure and lower VIS within 48 h after ICU admission. Additionally, it may be associated with an improved clinical course, represented by more ICU-free and vasopressor/inotrope-free days.

*Trial registration* UMIN Clinical Trial Registry on 1 November 2019 (registration no. UMIN000038302).

**Supplementary Information:**

The online version contains supplementary material available at 10.1186/s13054-025-05422-7.

## Background

Septic shock is the most severe form of infection complicated by profound circulatory failure; the mortality rate is as high as 30–40% [1, 2]. Hemodynamic management for circulatory failure using fluids and vasopressors is thought to be a crucial element of the management bundle for septic shock, and is associated with improved mortality [3, 4]. Early reversal of shock is the initial treatment goal to improve outcomes, as indicated by the elevation of mean arterial pressure and the subsequent tapering of vasopressors. Mean arterial pressure and vasopressor dosing during the first 24 h have been shown to be important factors for predicting mortality [5, 6].

Polymyxin B hemoperfusion (PMX-HP) is a treatment option to achieve hemodynamic stabilization by extracorporeal endotoxin removal. PMX-HP has been shown in randomized clinical trials to elevate mean arterial pressure and to decrease the dose of vasopressors in patients with septic shock [7, 8]. Early administration of PMX-HP may provide hemodynamic benefits by facilitating early reversal of shock, and it has been shown to be associated with a reduced dose and shortened duration of vasopressor therapy compared with late administration [9–12]. However, the detailed time course of hemodynamic parameters in relation to the timing of PMX-HP administration has not yet been described.

Regarding survival from septic shock, the reported results have been inconsistent. Early administration of PMX-HP has been shown in several small single-center observational studies to be associated with improved survival [9, 10, 12]. Conversely, no association between the timing of PMX-HP administration and mortality was shown in two larger multicenter observational studies [11, 13]. The reason for this discrepancy is unclear, but it might be due to the heterogeneity of patients' severity, differences in study design (e.g., multicenter vs. single-center), and variations in the methods of PMX-HP administration, including the duration: conventional (2 h) vs. prolonged (> 2 h). Although a randomized clinical trial is recommended when investigating the effect of an intervention, the current clinical circumstances made it difficult to conduct a randomized clinical trial to examine the timing of PMX-HP administration. Specifically, there were no clear indications for PMX-HP and there were ethical concerns in relation to patients with septic shock.

This multicenter observational study therefore examines the effect of the timing of PMX-HP administration among patients within a homogenously severe population. In this predetermined analysis, we examine the effects of the timing of PMX-HP administration on the short-term hemodynamics using the BEst Available Treatment for septic SHOCK (BEAT-SHOCK) registry. We also examine the association between the timing of PMX-HP administration and mortality.

## Methods

BEAT-SHOCK registry is a multicenter prospective registry that registered critically ill patients with septic shock requiring high-dose norepinephrine (≥ 0.2 μg/kg/min) from the ICUs of 20 hospitals in Japan between January 2020 and December 2022. The current study was approved by the Tohoku University Hospital Ethics Boards (approval number 2019-1-402), and was registered to the UMIN Clinical Trial Registry on 1 November 2019 (registration no. UMIN000038302). The Tohoku University Hospital Ethics Committee and the ethics committees of all other participating hospitals approved this study with an opt-out policy in accordance with the Ethical Guidelines for Medical and Biological Research Involving Human Subjects [14]. Written informed consent to collection of data about functional outcomes by the distribution of questionnaires at 90 days was obtained from patients or their proxies. This study was conducted in compliance with the Ethical Guidelines for Medical and Biological Research Involving Human Subjects and in accordance with the principles of the Declaration of Helsinki [14].

The inclusion criteria of the BEAT-SHOCK registry were adult patients (≥ 18 years old) with septic shock that were admitted to participating ICUs and who required high-dose norepinephrine (≥ 0.2 μg/kg/min) within 24 h after the onset of sepsis. The dose of norepinephrine was calculated as base-equivalent dose. Sepsis and septic shock were defined according to the Sepsis-3 criteria [2]. The registry excluded patients who died or were discharged from ICUs within 48 h after ICU admission, those who already had newly diagnosed organ dysfunction before the onset of sepsis, those with severe liver cirrhosis (Child–Pugh grade C), those with severe chronic heart failure (New York Heart Association classification for heart failure IV), those with acute myocardial infarction, and those who had received a cancer diagnosis with short life expectancy.

The BEAT-SHOCK registry is a multipurpose study registry designed to explore the effects of various managements against septic shock, including PMX-HP, in real-world settings. The sample size of this registry was set as 400 patients, which is based on the expected number of eligible patients during the study period. In this predetermined analysis, we included all the patients from the BEAT-SHOCK registry that were treated with PMX-HP during their ICU stay. We divided them into two groups (early and late administration groups) based on the median time from ICU admission to the initiation of PMX-HP. We focus here upon the subset cohort; the entire-cohort results of comparisons between patients in the BEAT-SHOCK registry treated with and without PMX-HP will be reported separately elsewhere.

In our study group, we administered PMX-HP for a prolonged duration (≥ 6 h) compared with the conventional duration (2 h). This approach was based on previous studies, which suggested that a prolonged duration of PMX-HP might provide sustained circulatory stabilization and improve mortality [15, 16]. The decision to administer PMX-HP and the timing of administration were at the discretion of the attending physicians.

The primary outcome was the short-term hemodynamic parameters within 48 h after ICU admission, including mean arterial pressure and vasoactive-inotropic score (VIS). VIS was calculated by the following equation [17]:

VIS = norepinephrine (μg/kg/min) × 100 + epinephrine (μg/kg/min) × 100 + dopamine (μg/kg/min) + dobutamine (μg/kg/min) + vasopressin (unit/kg/min) × 10,000 + levosimendan (μg/kg/min) × 50 + milrinone (μg/kg/min) × 10.

In our cohort, no patients received levosimendan or milrinone, so VIS was calculated from the doses of norepinephrine, epinephrine, dopamine, dobutamine, and vasopressin. Secondary outcomes included sequential organ failure assessment (SOFA) score on day 3, total amount of fluid within 48 h of ICU admission, ICU mortality, in-hospital mortality, 28 day and 90 day mortality, ICU-free days, and organ support-free days during the 28 days after ICU admission. Free days were calculated as 28 minus the number of days under the relevant state (e.g., ICU stay, mechanical ventilation, vasopressor/inotrope, and kidney replacement therapy). For patients who died within 28 days after ICU admission, each free day was assigned a value of zero (the worst value).

### Statistical analysis

Continuous variables are presented as medians and interquartile ranges (IQR), and categorical variables are presented as numbers and percentages (%). We evaluated the change in hemodynamic parameters from time 0 (starting PMX-HP) to each time point in all enrolled patients using the Wilcoxon signed rank test. For comparison between the two groups, the Wilcoxon rank-sum test was used for continuous variables, and Fisher’s exact test or the chi-square test was used for categorical variables as appropriate. We constructed univariate and multivariate Cox proportional hazard models to evaluate the association between the timing of PMX-HP (early and late administration) and survival time at 28 and 90 days. In the multivariate model, we used predefined adjusters selected based on previous literature and clinical judgement, which were age (≥ 65 years old), chronic illness (Charlson comorbidity index ≥ 3), disability (performance status ≥ 3), illness severity (acute physiology and chronic health evaluation II score ≥ 21), source of infection (urinary tract/abdomen or not), emergent surgery (yes/no), serum lactate concentration (≥ 4 mmol/L), VIS (≥ 30), and bacteremia (yes/no) [17–21]. There were no missing data pertinent to these adjusters. As a post-hoc sensitivity analysis, we compared three tertile groups based on the time from ICU admission to the administration of PMX-HP using the log-rank test for survival time at 90 days. Additionally, to adjust the severity on the day of PMX-HP initiation, we conducted a sensitivity analysis with a multivariate Cox proportional hazard model adjusting SOFA score on the day of PMX-HP initiation (dichotomized by the median value) instead of APACHE II score on the day of ICU admission. We also performed univariate and multivariate Cox proportional hazard models, with timing of treatment with PMX-HP as a continuous variable. Additionally, we conducted a receiver operating characteristic (ROC) curve analysis to explore the time to PMX-HP initiation other than the predetermined median cutoff. A two-sided *P* value < 0.05 was considered statistically significant, and all analyses were performed using JMP Pro Software (version 16.0.0; SAS Institute Inc., Cary, NC, USA).

## Results

**Fig. 1:**
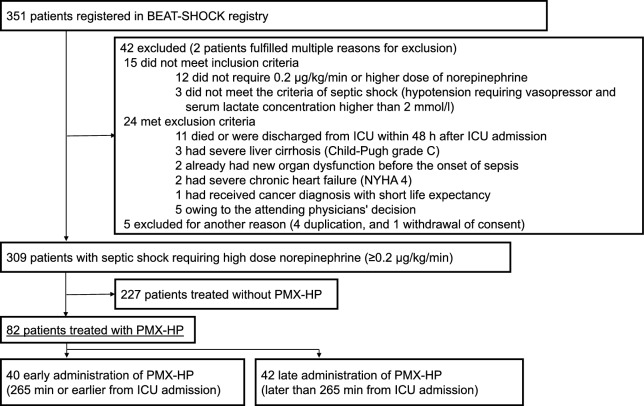
Patient flowchart. *BEAT-SHOCK* BEst Available Treatment for septic SHOCK, *ICU* intensive care units, *NYHA* New York Heart Association classification for heart failure, *PMX-HP* Polymyxin B hemoperfusion

Among the 309 patients with septic shock in the BEAT-SHOCK registry, 82 patients were treated with PMX-HP (27%) (Fig. 1). Median ages of patients treated with and without PMX-HP were 71 years old (interquartile range [IQR]: 62–80) and 73 years old (IQR: 65–81), respectively. Median SOFA scores were 11 (IQR: 9–13) and 11 (IQR: 9–14), respectively. Major sites of infection were the abdomen (37 patients [45%] and 78 patients [34%], respectively), the urinary tract (18 patients [22%] and 32 patients [14%], respectively), and the thorax (8 patients [10%] and 51 patients [23%], respectively) (Supplementary Table 1). The median time from ICU admission to the start of PMX-HP was 265 min (IQR: 113–480 min). We divided these 82 patients into the early administration group (time < 265 min, N = 40) and the late administration group (time ≥ 265 min, N = 42).

Patient characteristics are shown in Table 1. Patients in the early administration group had significantly lower scores of SOFA score on ICU admission than those in the late administration group (early group = 10 [IQR: 9–12] and late group = 12 [IQR: 9–13]; *P* = 0.025). On the day of PMX-HP initiation, the median SOFA scores were also lower in the early administration group than in the late administration group (10 [IQR: 9–12] and 12 [IQR: 10–14]; *P* = 0.014). Details related to PMX-HP are presented in Table 2. The duration of the first session of PMX-HP was 1035 min (IQR: 518–1331 min) in the early administration group and 995 min (IQR: 553–1390 min) in the late administration group (*P* = 0.93). A second session of PMX-HP was received by 17 (43%) and 19 (45%) patients in the early and late administration groups, respectively.

**Table 1:** Baseline demographics and clinical characteristics

Characteristics	Early administration group (n = 40)	Late administration group (n = 42)	*P* value
Age, y, median (IQR)	70 (59–81)	72 (64–80)	0.77
Male, n (%)	21 (53)	25 (60)	0.52
Body mass index, median (IQR)	23.4 (20.8–31.1)	23.0 (20.5–26.5)	0.34
Route of ICU admission			0.95
Emergency department, n (%)	31 (78)	31 (74)	
General wards, n (%)	1 (3)	2 (5)	
Other ICU, n (%)	1 (3)	1 (2)	
Other hospital, n (%)	7 (18)	8 (19)	
Emergent surgery, n (%)	19 (48)	17 (40)	0.52
SOFA score on ICU admission, median (IQR)	10 (9–12)	12 (9–13)	0.025
APACHE II score on ICU admission, median (IQR)	24 (20–30)	28 (21–32)	0.16
Comorbidity^a^			
Chronic hemodialysis, n (%)	3 (8)	1 (2)	0.35
Immuno-compromised, n (%)	3 (8)	1 (2)	0.35
Chronic respiratory disorder, n (%)	2 (5)	1 (2)	0.61
Liver cirrhosis, n (%)	0 (0)	0 (0)	1
Chronic heart failure, n (%)	0 (0)	0 (0)	1
Charlson comorbidity index, median (IQR)	2 (0–4)	1 (0–3)	0.98
Performance status prior to hospitalization, n (%)^b^			0.30
Normal activity, n (%)	26 (65)	18 (43)	
Restricted in strenuous activity, n (%)	9 (23)	12 (29)	
Ambulatory but unable to carry out any work activities, n (%)	2 (5)	6 (14)	
Stay in a chair or in bed for more than 50% of the day, n (%)	2 (5)	4 (10)	
Completely disabled and bedridden, n (%)	1 (3)	2 (5)	
Site of infection			0.53
Abdomen, n (%)	21 (53)	16 (38)	
Urinary tract, n (%)	8 (20)	10 (24)	
Thorax, n (%)	2 (5)	6 (14)	
Skin and soft tissue, n (%)	7 (18)	7 (17)	
Others, n (%)	2 (5)	3 (7)	
Isolated pathogen			0.40
Gram-negative bacteria, n (%)	21 (53)	18 (43)	
Gram-positive bacteria, n (%)	3 (8)	7 (17)	
Others (including unknown), n (%)	16 (40)	17 (41)	
Lactate concentration on ICU admission, mmol/L, median (IQR)	4.0 (2.4–6.3)	4.8 (3.0–7.0)	0.098
Mean arterial pressure on ICU admission, mmHg, median (IQR)	70 (60–82)	71 (60–81)	0.11
Heart rate on ICU admission, bpm, median (IQR)	111 (86–136)	104 (93–120)	0.73
Treatment on the day of ICU admission			
Maximum norepinephrine dosage within 6 h after ICU admission, μg /kg/min, median (IQR)	0.32 (0.24–0.47)	0.30 (0.20–0.34)	0.10
Maximum vasopressor-inotrope score within 6 h after ICU admission, median (IQR)^d^	42.6 (29.7–58.3)	35.9 (25.9–43.7)	0.059
Corticosteroid administration, n (%)	35 (88)	34 (81)	0.41
Invasive mechanical ventilation, n (%)	31 (78)	32 (76)	0.89
Continuous kidney replacement therapy, n (%)	25 (63)	26 (62)	0.96

*IQR* Interquartile range, *ICU* intensive care unit, *SOFA* sequential organ failure assessment, *APACHE II* Acute Physiology and Chronic Health Evaluation II

^a^Comorbidity was defined in accordance with APACHE II score definition

^b^Performance status was classified according to World Health Organization performance status classification

^c^Treatment on the day of ICU admission includes the treatment on the first and second calendar days of ICU admission, except for norepinephrine and vasopressor-inotrope score

^d^Vasopressor-inotrope score = dopamine (μg/kg/min) + dobutamine (μg/kg/min) + 100 × epinephrine (μg/kg/min) + 100 × norepinephrine (μg/kg/min) + 10 × milrinone (μg/kg/min) + 10,000 × vasopressin (units/kg/min) + 50 × levosimendan (μg/kg/min)

**Table 2:** Details of polymyxin B hemoperfusion

Characteristics	Early administration group (n = 40)	Late administration group (n = 42)	*P* value
Time from ICU admission to the first session of PMX-HP, min, median (IQR)	110 (60–180)	465 (328–748)	< 0.0001
Duration of the first session of PMX-HP, min, median (IQR)	1035 (518–1331)	995 (553–1390)	0.93
Blood flow rate during the 1st session of PMX-HP, ml/min, median (IQR)	100 (80–100)	100 (80–100)	0.42
Anticoagulation for hemoperfusion during first session of PMX-HP			0.56
Nafamostat mesylate, n (%)	38 (95)	38 (90)	
Unfractionated heparin, n (%)	2 (5)	3 (7)	
None, n (%)	0 (0)	1 (2)	
Premature interruption due to circuit clotting during the first session of PMX-HP, n (%)	15 (38)	10 (24)	0.23
Implementation of the second session of PMX-HP, n (%)	17 (43)	19 (45)	0.83
Time from ending the first session to starting the second session of PMX-HP, min, median (IQR)^a^	110 (18–384)	115 (10–246)	0.66
Duration of second session of PMX-HP, min, median (IQR)^a^	840 (514–1485)	1290 (1187–1566)	0.16

*ICU* intensive care unit, *PMX-HP* polymyxin B hemoperfusion, *IQR* Interquartile range

^a^Data about patients received the second session of PMX-HP (17 patients in the early administration group and 19 patients in the late administration group)

In all of the 82 enrolled patients, mean arterial pressure significantly increased at 4 h and thereafter from the time of starting PMX-HP (Supplementary Fig. 1). Similarly, VIS in all 82 patients significantly decreased at 4 h and thereafter from the time of starting PMX-HP (Supplementary Fig. 2).

**Fig. 2:**
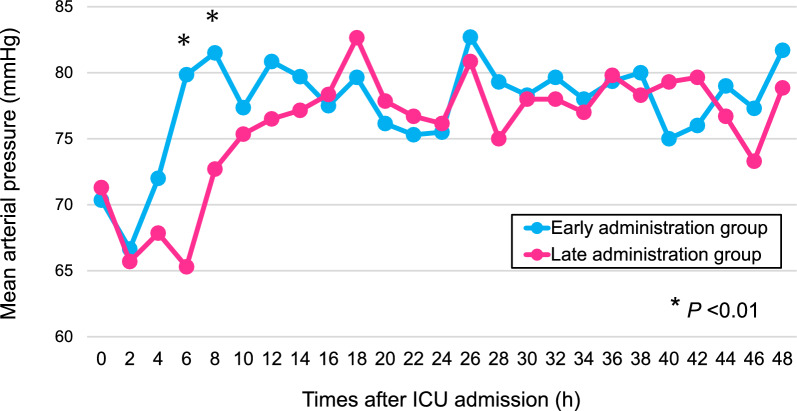
Mean arterial pressure between early and late administration groups within 48 h after ICU admission. At 6 h and 8 h after ICU admission, mean arterial pressures were significantly higher in the early administration group. We used Wilcoxon rank sum test for each comparison between the early and late administration groups and the median values for mean arterial pressure are shown. *ICU* intensive care unit

**Fig. 3:**
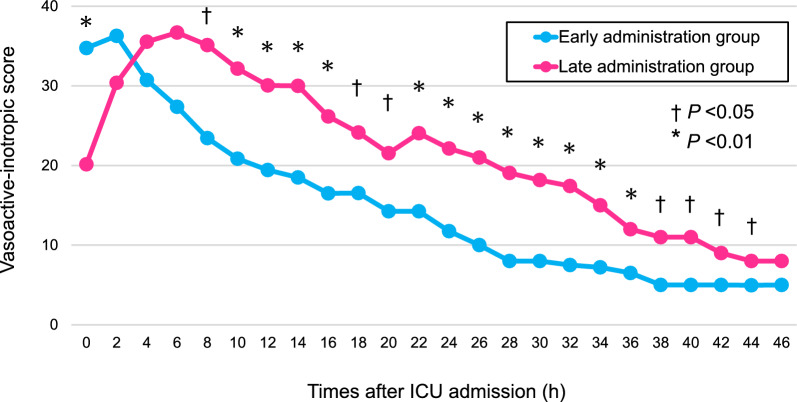
Vasoactive-inotropic score between the early and late administration groups within 48 h after ICU admission. At 8 h and thereafter from ICU admission, vasoactive-inotropic score was significantly lower in the early administration group. Wilcoxon rank sum test was used for each comparison between the early and late administration groups. Figure shows the median values for vasoactive-inotropic score. *ICU* intensive care unit

When we compared mean arterial pressures between the early and late administration groups, mean arterial pressures increased earlier in the early administration group than in the late administration group and were significantly higher at 6 h and 8 h after ICU admission (Fig. 2). Regarding VIS, the VIS in early administration group decreased earlier than in the late administration group (Fig. 3). At 8 h after ICU admission and thereafter, VIS was significantly lower in the early administration group.

**Fig. 4:**
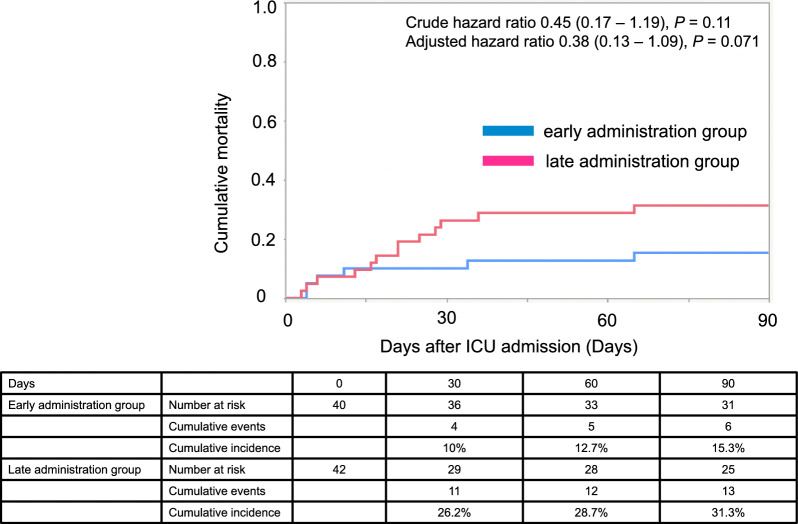
Ninety-day mortality after ICU admission in the early and late administration groups. Time-to-event curve for death at 90 days after ICU admission is shown. The hazard ratio is from unadjusted and adjusted analyses by Cox proportional hazard model. In the adjusted model, we used predefined adjusters as age (≥ 65 years old), chronic illness (Charlson comorbidity index ≥ 3), disability (performance status ≥ 3), illness severity (acute physiology and chronic health evaluation II score ≥ 21), source of infection (urinary tract/abdomen or not), emergent surgery (yes/no), serum lactate concentration (≥ 4 mmol/L), vasoactive-inotropic score (≥ 30), and bacteremia (yes/no)

The secondary outcomes are presented in Table 3. ICU-free days and vasopressor/inotrope-free days were significantly longer in the early administration group. The 28-day mortality rate was 10% in the early administration group and 24% in the late administration group (*P* = 0.097). In the survival time analysis for 28-day mortality, the unadjusted hazard ratio for the early administration group was 0.41 (95% confidence interval [CI] 0.13–1.30, *P* = 0.13), and the adjusted hazard ratio was 0.30 (95%CI 0.083–1.09; *P* = 0.067). Similar results were observed for 90-day mortality analysis including the sensitivity analysis (Fig. 4, Supplementary Fig. 3, Supplementary Table 2, 3). ROC curve analysis of the time to PMX-HP initiation and 28-day mortality showed that the 675 min was the cutoff point based on the Youden-index metric but the median value (265 min) was close to the diagonal line (Supplementary Fig. 4). We thus constructed the additional Cox-proportional hazard model as a sensitivity analysis with the Youden-index cutoff point (675 min), which yielded similar results for 28-day mortality.

**Table 3:** Secondary outcomes

Outcomes	Early administration group (n = 40)	Late administration group (n = 42)	*P* value
Change of vasopressor-inotrope score from 0 to 48 h of ICU admission, median (IQR)	− 31.0 (− 41.9–− 23.8)	− 23.4 (− 32.4–− 10.7)	0.0047
Change of sequential organ failure assessment score from day 1 to day 3 of ICU admission, median (IQR)	0 (− 2–3)	2 (− 1–3)	0.39
Total amount of fluid within 48 h of ICU admission, ml, median (IQR)	8404 (6527–10,946)	7357 (6098–10565)	0.70
ICU-free days during 28 days, days, median (IQR)	18 (1–23)	14 (0–19)	0.025
Ventilator-free days during 28 days, days, median (IQR)	22 (6–25)	19 (0–23)	0.081
Vasopressor/inotrope-free days during 28 days, days, median (IQR)	23 (21–25)	21 (0–24)	0.027
Kidney replacement therapy-free days during 28 days, days, median (IQR)	25 (22–28)	25 (0–28)	0.39
28 day mortality, n (%)	4 (10)	10 (24)	0.097
ICU mortality, n (%)	4 (10)	9 (21)	0.16
In-hospital mortality, n (%)	6 (15)	13 (31)	0.087

*ICU* intensive care unit, *IQR* Interquartile range

## Discussion

After starting PMX-HP, mean arterial pressure increased, and VIS decreased from 4 h and thereafter among patients with refractory septic shock in real-world settings. Early administration of PMX-HP (< 265 min after ICU admission) was associated with improved hemodynamic parameters compared with late administration (≥ 265 min), including early restoration of mean arterial pressure and subsequent tapering of vasopressors and inotropes. Regarding the clinical course, early administration was associated with more vasopressor/inotrope-free days and more ICU-free days. Although it did not reach statistical significance due to the small sample size, the early administration group had a numerically lower cumulative 90-day mortality rate than the late administration group (15.3% vs. 31.3%).

Regarding the hemodynamic effect of PMX-HP, a previous randomized clinical trial enrolled 64 patients with sepsis that underwent emergent surgery. Mean arterial pressure reportedly increased from 76 mmHg at baseline to 84 mmHg at 72 h, and the vasopressor requirement decreased significantly in the PMX-HP group [7]. Another randomized controlled trial enrolled 450 patients with septic shock and elevated endotoxin activity. Change in mean arterial pressure from baseline to day 3 was significantly higher in the PMX-HP group than that in the sham group (9.4 vs. 4.1 mmHg) [8]. Similarly, our study demonstrated that hemodynamic parameters, represented by mean arterial pressure and VIS, improved significantly at 4 h after starting PMX-HP, and this improvement was sustained at 24 h.

Regarding the timing of PMX-HP administration, several studies have reported an association between early administration and hemodynamic improvement compared with late administration [9, 11]. A retrospective study showed that more patients had lowering of catecholamines 7 days after PMX-HP in early administration group [11]. In another study, the catecholamine index was significantly lower 24 h after PMX-HP in the early administration group than in the late administration group [9]. In this context, our study detailed the time course of hemodynamic parameters between the early and late administration groups. We observed a significant elevation of mean arterial pressure at 6 h and 8 h after ICU admission, followed by the tapering of vasopressors. Early administration of PMX-HP is implied to have led to early restoration of hemodynamic stabilization in patients with septic shock.

The association between the timing of PMX-HP administration and mortality has differed in previous reports. Three single-center retrospective studies, for example, showed that the mortality rate was significantly lower in patients who received PMX-HP early compared with those who received it later [9, 10, 12]. In these studies, PMX-HP was applied for a more prolonged duration (12–24 h) than the conventional duration (2 h). Conversely, two larger multicenter retrospective studies found no association between the timing of PMX-HP administration and mortality [11, 13], but PMX-HP was administered for the conventional 2 h duration. This discrepancy might result from differences in study design (single-center vs. multicenter) and the duration of PMX-HP (prolonged duration vs. 2 h). The early administration of PMX-HP over prolonged durations might improve clinical outcomes, but the results from the abovementioned single-center studies must to be reproduced in multicenter studies. In our study, PMX-HP was predominantly administered for prolonged durations, with a median duration of approximately 17 h. This prolonged duration may have contributed to the sustained hemodynamic stabilization observed in patients that received early administration of PMX-HP.

Early hemodynamic stabilization by the early administration of PMX-HP over a prolonged period might improve the clinical course. In our findings, vasopressor/inotrope-free days and ICU-free days were improved in the early administration group compared with in the late administration group. In critically-ill patients, these vasopressor/inotrope-free days and ICU-free days were thought to be an important surrogate outcome for long-term mortality. Correlation between organ support-free days including vasopressor usage and 180-day survival outcomes has been reported [22]. In our study, the 90-day cumulative mortality rate was lower in the early administration group, although without statistical significance. A clinically significant effect on mortality cannot be excluded due to the wide ranging confidence interval. Future trials examining the effect of PMX-HP on mortality should consider both the timing and the duration of PMX-HP administration as important factors that could affect the outcomes. When clinicians judge PMX-HP to be necessary for the treatment of septic shock, we suggest it should be administered as early as possible to achieve early hemodynamic stabilization.

This study has several limitations. First, the sample size was too small to conclude the mortality effect of the timing of PMX-HP administration. However, we applied a rigorous study design, including a multicenter prospective approach and multivariate analysis to adjust for predefined confounders. Our mortality results suggest the need for further investigation in future studies.

Second, we divided patients into two groups using the median value (265 min) for the timing of PMX-HP administration, rather than using a predefined cutoff. A ROC curve analysis suggested that 675 min could be used for the cutoff value of the time to PMX-HP initiation. The meaning of the median cutoff value requires careful interpretation, but it is similar to those used in previous studies (ranging from 3–9 h) [9–12]. We suggest the 265 min cutoff can therefore be considered to be a clinically meaningful threshold.

Third, the nature of observational study design could not demonstrate a causal relationship due to certain factors, especially confounders, lead-time bias, and immortal-time bias. For example, patients in the late administration group had higher SOFA scores than those in the early administration group. Patients with less severe conditions might have received PMX-HP earlier, for example, which could have confounded the results. Our findings should therefore be considered exploratory and hypothesis-generating and confirmation in future studies is required. However, we suggest the influence of residual confounders might be minimal because we carefully selected predefined adjusters, including disease severity scores from clinical experiences and from existing literature, and we prospectively collected the data to construct the multivariate models. Regarding lead-time bias, patients in the late administration group received hemodynamic management other than PMX-HP for a longer time before starting PMX-HP than those in the early administration group. Indeed, the VIS in the late administration group worsened within the first 6 h after ICU admission, suggesting differences in the indication for PMX-HP (Fig. 3). Furthermore, patients starting PMX-HP later had shorter treatment time within the first 48 h after ICU admission, which might attenuate the short-term hemodynamic effect of PMX-HP. However, patients in the early administration group had a better clinical course beyond short-term hemodynamic parameters, including more vasopressor/inotrope-free and ICU-free days. Theoretically, short-term hemodynamic parameters were susceptible to lead-time bias; however, differences in the clinical course suggested that the differences in these parameters were due to the timing of PMX-HP initiation rather than solely the result of lead-time bias. Furthermore, sensitivity analysis adjusting the severity at the time of PMX-HP initiation showed consistent results with primary analysis. As for immortal-time bias, patients in the late administration group may have longer survival times than those in the early administration group, potentially affecting the results of the survival time analysis. However, the median time from ICU admission to starting the PMX-HP in the late administration group was 465 min (IQR: 328–748), and our study excluded patients who died within 48 h after ICU admission. Most patients in the late administration group were therefore unaffected by immortal-time bias.

## Conclusions

In patients with septic shock, early administration of PMX-HP was associated with higher mean arterial pressure and lower VIS within 48 h after ICU admission. Early administration may be also associated with an improved clinical course, as shown by more ICU-free and vasopressor/inotrope-free days.

## Supplementary Information

Supplementary material 1.

## Data Availability

No datasets were generated or analysed during the current study.

## Abbreviations

BEAT-SHOCK: The BEst available treatment for septic SHOCK registry
ICU: Intensive care unit
IQR: Interquartile ranges
PMX-HP: Polymyxin B hemoperfusion
ROC: Receiver operating characteristic
SOFA: Sequential organ failure assessment
VIS: Vasoactive-inotropic score
