# Supplementary material for: Time to administer polymyxin B hemoperfusion and hemodynamics in patients with septic shock requiring high-dose norepinephrine: a predetermined analysis of a prospective cohort study
Source: Crit Care. 2025 May 9;29:187. doi: 10.1186/s13054-025-05422-7 (PMC12063383; doi:10.1186/s13054-025-05422-7)
Supplement: Supplementary file 1 — Supplementary material 1. [file 13054_2025_5422_MOESM1_ESM.pdf]

Additional files

- Supplementary Figure 1. Mean arterial pressure before and after starting polymyxin B hemoperfusion
- Supplementary Figure 2. Vasoactive-inotropic score before and after starting polymyxin B hemoperfusion
- Supplementary Figure 3. Ninety-day mortality after ICU admission in each tertile group
- Supplementary Figure 4. Receiver operating characteristic curve analysis of the time to PMX-HP initiation and 28-day mortality.

Additional files

- Supplementary Table 1. Baseline demographics and clinical characteristics in patients treated with and without PMX-HP from entire cohort of BEAT-SHOCK registry .
- Supplementary Table 2. Hazard ratio of time from ICU admission to administration of PMX-HP for 90-day mortality adjusted by SOFA score on the day of PMX-HP initiation.
- Supplementary Table 3. Hazard ratio of time from ICU admission to administration of PMX-HP as a continuous variable for 90-day mortality.

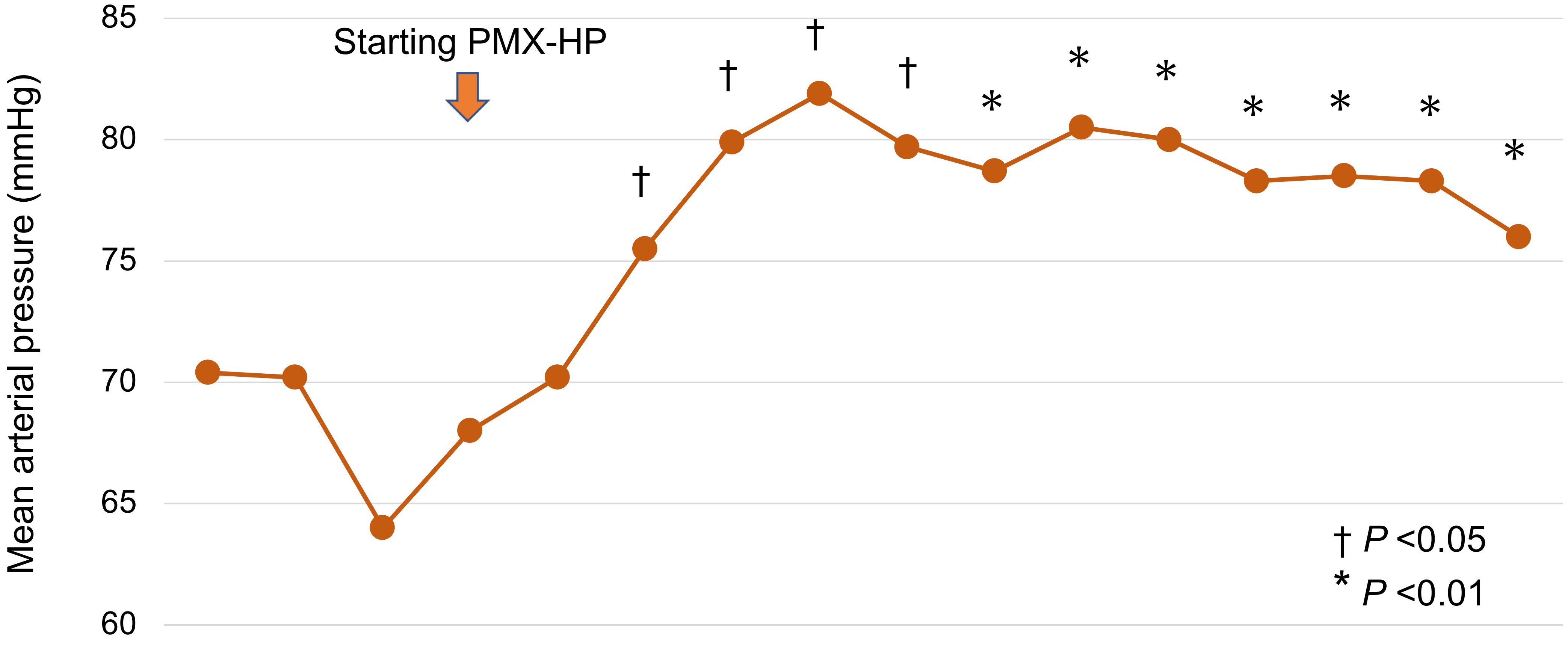

Hours from starting PMX-HP	-6	-4	-2	0	2	4	6	8	10	12	14	16	18	20	22	24
Number of patients	30	48	61	81	82	82	82	82	81	79	80	80	79	80	77	78

Supplementary Figure 1. Mean arterial pressure before and after starting polymyxin B hemoperfusion
At four hours and thereafter from starting polymyxin B hemoperfusion, mean arterial pressure significantly elevated from 0 hour. We used signed Wilcoxon rank sum test for each comparison between mean arterial pressures at 0 hour and at each time point. Median values for mean arterial pressure are shown.
PMX-HP; polymyxin B hemoperfusion.

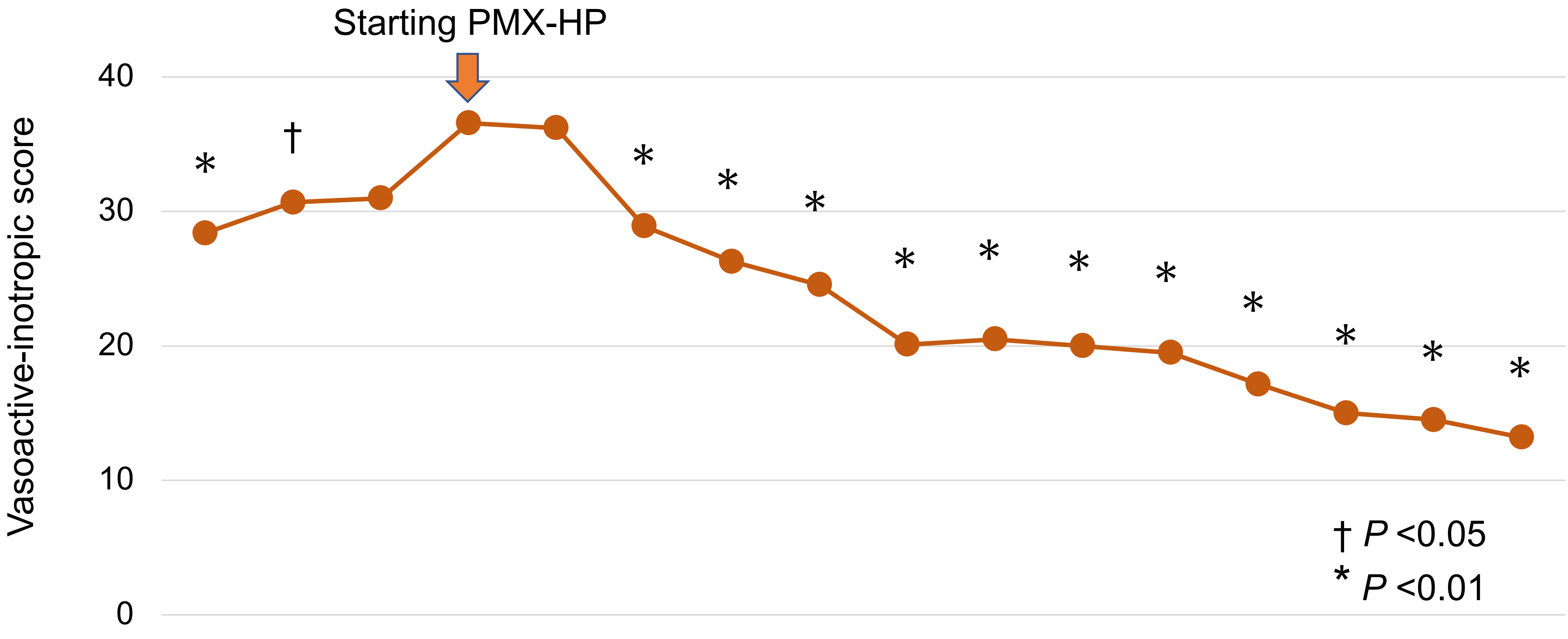

Hours from starting PMX-HP	-6	-4	-2	0	2	4	6	8	10	12	14	16	18	20	22	24
Number of patients	30	48	61	82	82	82	82	81	80	80	80	80	80	79	78	76

Supplementary Figure 2. Vasoactive-inotropic score before and after starting polymyxin B hemoperfusion

At four hours and thereafter from starting polymyxin B hemoperfusion, vasoactive-inotropic score (VIS) significantly decreased from 0 hour. We used signed Wilcoxon rank sum test for each comparison between VIS at 0 hour and at each time point. Median values for VIS are shown. VIS was calculated as: norepinephrine (µg/kg/min) x 100 + epinephrine (µg/kg/min) x 100 + dopamine (µg/kg/min) + dobutamine (µg/kg/min) + vasopressin (unit/kg/min) x 10,000 + levosimendan (µg/kg/min) x 50 + milrinone (µg/kg/min) x 10.

PMX-HP; polymyxin B hemoperfusion.

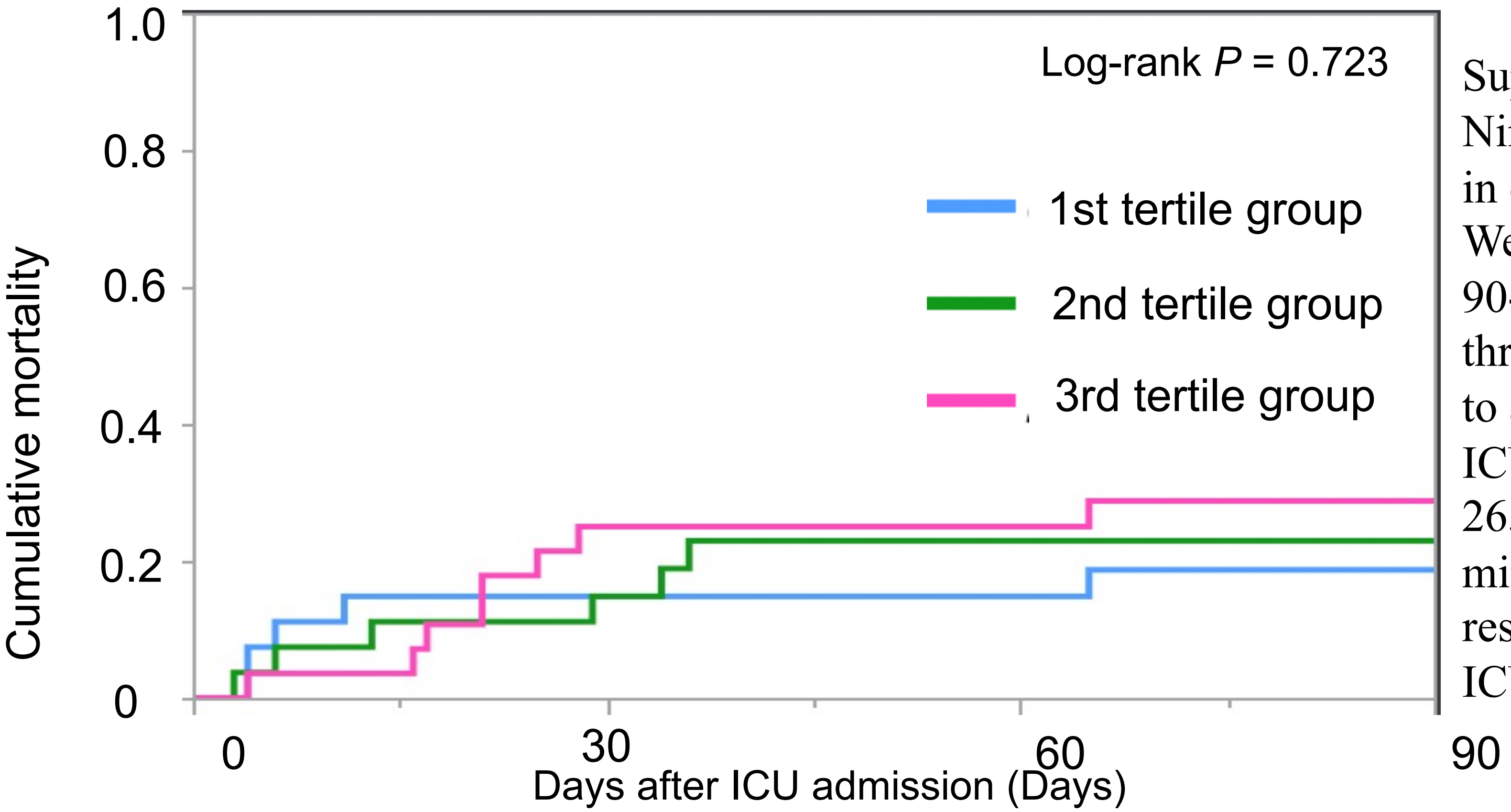

Supplementary Figure 3.
Ninety-day mortality after ICU admission in each tertile group.
We present time-to-event curve for death at 90-days after ICU admission. We divided into three tertile groups by time from ICU admission to administration of PMX-HP. Median time from ICU admission to PMX-HP were 65 (IQR 50–115), 265 (IQR 230–310), and 690 (IQR 463–955) minutes in the 1st, 2nd, and 3rd tertile groups, respectively.
ICU: intensive care unit; IQR: interquartile range; PMX-HP: polymyxin B hemoperfusion.

Days		0	30	60	90
1st tertile	Number at risk	27	24	23	20
	Cumulative events		4	4	5
	Cumulative incidence		14.8%	14.8%	18.7%
2nd tertile	Number at risk	27	22	20	19
	Cumulative events		4	6	6
	Cumulative incidence		14.8%	22.9%	22.9%
3rd tertile	Number at risk	28	20	20	17
	Cumulative events		7	7	8
	Cumulative incidence		25.0%	25.0%	28.8%

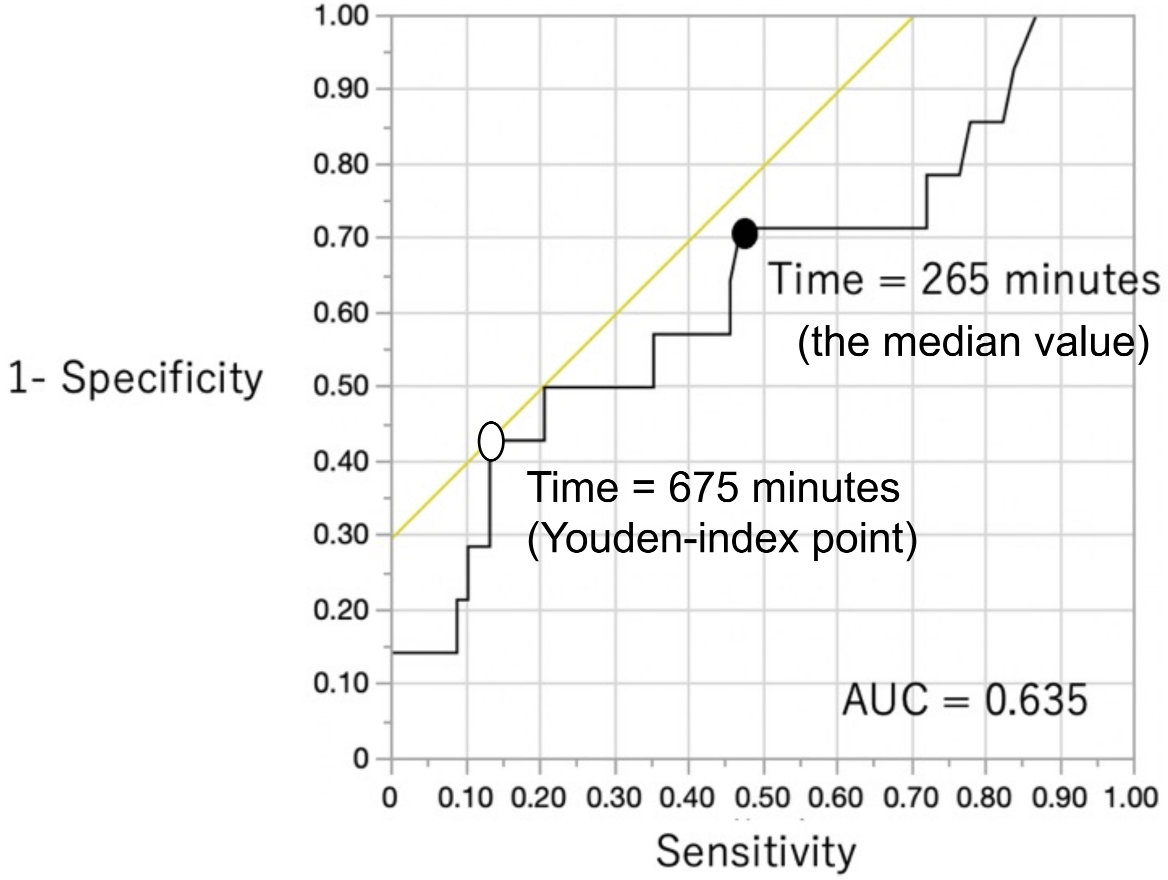

Supplementary Figure 4. Receiver operating characteristic curve analysis of the time to PMX-HP initiation and 28-day mortality.

The median time of 265 min (black dot) was close to the Youden-index metrics (diagonal line). Cox proportional hazard models using the Youden-index cutoff point (675 min) showed a crude hazard ratio of 0.35 (95%CI 0.12–1.06) and an adjusted hazard ratio of 0.20 (95%CI 0.05–0.79) for 28-day mortality, comparing early initiation with late initiation.

PMX-HP: polymyxin B hemoperfusion; CI: confidence interval; AUC: area under the curve.

Supplementary Table 1. Baseline demographics and clinical characteristics in patients treated with and without PMX-HP from entire cohort of BEAT-SHOCK registry

	Patients treated with PMX-HP (n = 82)	Patients treated without PMX-HP (n = 227)
Age, y, median (IQR)	71 (62–80)	73 (65–81)
Male, n (%)	46 (56)	129 (57)
Emergent surgery, n (%)	36 (44)	72 (32)
SOFA score at ICU admission, median (IQR)	11 (9–13)	11 (9–14)
APACHE II score on ICU admission, median (IQR)	26 (21–31)	27 (22–33)
Comorbidity*		
Chronic hemodialysis, n (%)	4 (5)	15 (7)
Immuno-compromised, n (%)	4 (5)	11 (5)
Chronic respiratory disorder, n (%)	3 (4)	3 (1)
Liver cirrhosis, n (%)	0 (0)	5 (2)
Chronic heart failure, n (%)	0 (0)	0 (0)
Charlson comorbidity index, median (IQR)	1 (0–3)	1 (0–3)
Site of infection		
Abdomen, n (%)	37 (45)	78 (34)
Urinary tract, n (%)	18 (22)	32 (14)
Thorax, n (%)	8 (10)	51 (23)
Skin and soft tissue, n (%)	14 (17)	42 (19)
Others, n (%)	5 (6)	24 (11)
Isolated pathogen		
Gram-negative bacteria, n (%)	39 (48)	96 (42)
Gram-positive bacteria, n (%)	10 (12)	40 (18)
Others (including unknown), n (%)	33 (40)	91 (40)
Lactate concentration on ICU admission, mmol/L, median (IQR)	4.2 (2.7–6.7)	4.0 (2.6–6.5)
Mean arterial pressure on ICU admission, mmHg, median (IQR)	71 (60–81)	68 (56–79)
Heart rate on ICU admission, bpm, median (IQR)	108 (90–127)	110 (95–124)
Maximum norepinephrine dosage within 6 h after ICU admission, μg/kg/min, median (IQR)	0.30 (0.24–0.43)	0.28 (0.20–0.40)

* Comorbidity was defined in accordance with the APACHE II score definition.

PMX-HP: polymyxin B hemoperfusion; BEAT-SHOCK: BEst Available Treatment for septic SHOCK; IQR: interquartile range; ICU: intensive care unit; SOFA: sequential organ failure assessment; APACHE II: Acute Physiology and Chronic Health Evaluation II.

Supplementary Table 2. Hazard ratio of time from ICU admission to administration of PMX-HP for 90-day mortality adjusted by SOFA score on the day of PMX-HP initiation.

	crude HR (95%CI)	*P* value	adjusted HR (95%CI)	*P* value
Time from ICU admission to administration of PMX-HP, early vs. late	0.45 (0.17–1.19)	0.11	0.54 (0.20–1.51)	0.24

We constructed a multivariate Cox proportional hazard model to evaluate the association between the timing of PMX-HP (early vs. late administration) and survival time at 90 days. In this post-hoc multivariate model, we used SOFA score on the day of PMX-HP initiation as an adjuster instead of APACHE II score on the day of ICU admission to adjust the severity on the day of PMX-HP initiation. Other adjusters were identical with primary analysis, which were age (≥65 years old), chronic illness (Charlson comorbidity index ≥3), disability (performance status ≥3), source of infection (urinary tract/abdomen or not), emergent surgery (yes/no), serum lactate concentration (≥4 mmol/L), vasoactive inotropic score (≥30), and bacteremia (yes/no).

ICU: intensive care unit; HR: hazard ratio; CI: confidence interval; PMX-HP: polymyxin B hemoperfusion; SOFA: sequential organ failure assessment; APACHE: acute physiology and chronic health evaluation.

Supplementary Table 3. Hazard ratio of time from ICU admission to administration of PMX-HP as a continuous variable for 90-day mortality.

	crude HR (95%CI)	*P* value	adjusted HR (95%CI)	*P* value
Time from ICU admission to administration of PMX-HP, h	1.04 (0.99–1.08)	0.071	1.05 (1.00–1.09)	0.057

We constructed univariate and multivariate Cox proportional hazard models to evaluate the association between the timing of PMX-HP as continuous variable (h) and survival time at 90 days. In the multivariate model, we used predefined adjusters selected based on previous literature and clinical judgement, which were age (≥65 years old), chronic illness (Charlson comorbidity index ≥3), disability (performance status ≥3), illness severity (acute physiology and chronic health evaluation II score ≥21), source of infection (urinary tract/abdomen or not), emergent surgery (yes/no), serum lactate concentration (≥4 mmol/L), vasoactive inotropic score (≥30), and bacteremia (yes/no).
ICU: intensive care unit; HR: hazard ratio; CI: confidence interval; PMX-HP: polymyxin B hemoperfusion.
